# Familial adenomatous polyposis associated with desmoid tumors presenting with abdominal abscess: A case report and literature review

**DOI:** 10.1097/MD.0000000000027897

**Published:** 2021-11-19

**Authors:** Ailing Liu, Hua Liu, Xueli Ding, Jun Wu, Zibin Tian, Tao Mao

**Affiliations:** Department of Gastroenterology, the Affiliated Hospital of Qingdao University, Qingdao, Shandong Province, China.

**Keywords:** aggressive fibromatosis, desmoid tumors, familial adenomatous polyposis

## Abstract

**Rationale:**

Familial adenomatous polyposis (FAP) associated with desmoids tumors (DTs) complicated by abscess formation is rare. The management is not easy and the choice of the best treatment may be controversial.

**Patient concerns:**

A 33-year-old man was admitted to our hospital for abdominal pain, fever, chills, nausea, and vomiting. He had a family history of FAP, and history of abdominal surgery.

**Diagnoses:**

An abdominal enhanced chest computed tomography (CT) scan revealed a soft tissue mass in the abdominal wall and an irregular mesenteric soft tissue mass with internal fistula and intra-abdominal abscess. A CT-guided biopsy of the abdominal wall mass revealed DTs.

**Interventions:**

The patient was given oral antibiotics for 6 months, and ultimately underwent surgery.

**Outcomes:**

The patient had no evidence of recurrence on follow-up at 10 months.

**Lessons:**

This case indicates that for patients with FAP who have a history of abdominal surgery and a progressively enlarging mass and abscess in the abdomen, it is necessary to consider the possibility of DTs. FAP-related DTs are rarely complicated by abscess formation. Antibiotic therapy plus surgical resection of the tumor may be effective and make good prognosis.

## 1 Introduction

Familial adenomatous polyposis (FAP) is a cancer syndrome caused by germline mutations in the adenomatous polyposis coli gene.^[1]^ It is characterized by the presence of hundreds of colonic polyps, which have a high tendency to undergo malignant transformation. Among the types of lesions associated with FAP, desmoid tumors (DTs) are potentially life-threatening that require special attention.^[2]^ DTs are rare and easy to miss in diagnosis. Most patients with DTs are asymptomatic and have chronic progression, resulting in a solid abdominal mass which might present with abdominal pain. Complications of DTs result from their locally aggressive nature, causing compression and invasion of the adjacent structures. Although intra-abdominal DTs may cause intestinal obstruction, bleeding, and perforation, it rarely forms an abscess. To our knowledge, there are few reports about of FAP-related DTs complicated with intra-abdominal abscess.^[3]^ Here, we report the first case of FAP-related DTs initially presented with intra-abdominal abscess in China. The patient was successfully treated with antibiotics and ultimately underwent surgery. He was doing well at the last follow-up of 10 months.

## 2 Case report

A 33-year-old male patient was admitted to our hospital in February 2020 due to acute lower abdominal pain, fever, nausea, and vomiting in the absence of diarrhea, hematochezia, cough, and sputum. The patient had a history of FAP for 2 years. He had a surgical history consisting of an appendectomy, cholecystectomy, and colon polypectomy. A laparotomy was performed for intestinal obstruction in 2018, where a cystic mass at the mesentery root was found and resected, but the pathology was unknown. The patient's mother died of colon cancer. His personal history was unremarkable. A physical examination upon admission: Several surgical scars were visible on the abdomen. A hard mass of about 10 cm × 10 cm in the left abdomen was palpable and immovable with pressure pain. Bowel sounds were normal. The lower limbs were not swollen.

After admission, the patient underwent thorough evaluations. The white blood cell count was 10.33 × 10^9^/L and the neutrophil count was 7.75 × 10^9^/L. The C-reactive protein level was 235.66 mg/L. Serum albumin was 29.2 g/L. Serum procalcitonin was 0.261 ng/mL. Common serum tumor markers, such as alpha-fetoprotein, carbohydrate antigen 19-9, carcinoembryonic antigen, and carbohydrate antigen 125 were normal. An abdominal enhanced chest computed tomography (CT) scan (February 2020, Fig. 1A) revealed a soft tissue mass in the abdominal wall and an irregular mesenteric soft tissue mass, with internal fistula and abdominal abscess. A CT-guided biopsy of the abdominal wall mass revealed DTs. Immunohistochemistry of the biopsy determined the sample to be β-Catenin nucleoplasm (+), CD34 (-), CD117 (-), Dog-1 (-), CD99 (-), STAT6 (-), SMA (-), S100 (-), ALK (5A4) (+), and MUC-4 (-).

**Figure 1:**
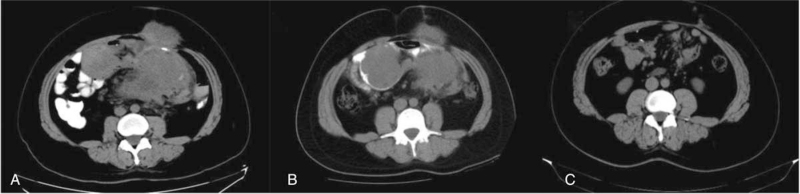
(A) The abdominal enhanced CT scan (February 2020) showed a soft tissue mass in the abdominal wall and an irregular mesenteric soft tissue mass with internal fistula. (B) The abdominal enhanced CT scan (August 2020) showed intraperitoneal abscess was smaller than before. The abdominal wall soft tissue mass was the same as before. There were multiple irregular nodules in the mesentery and multiple small lymph nodes behind the peritoneum. (C) The abdominal enhanced CT scan (July 2021) showed there were abdominal wall scars and high-density staples, and the anastomotic wall was slightly thickened. CT = chest computed tomography.

**Figure 2:**
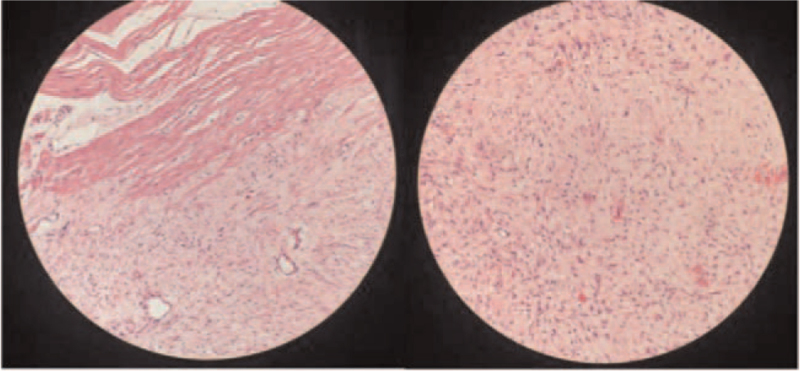
The pathology was spindle cell tumor with unclear borders, which invaded the surrounding fat and skeletal muscle tissue, involving the plasma layer and muscular layer of partial small intestine, in line with AF (HE 200×). AF = aggressive fibromatosis.

The patient was diagnosed with FAP-related DTs complicated with intra-abdominal abscess. He was initially treated with imipenem. The abdominal pain decreased, and the body temperature returned to normal. Due to the large size of the abdominal mass, it could not be totally resected and therefore surgery was not performed immediately. The patient was treated with levofloxacin for 6 months. Reinspection by abdominal enhanced CT scan (August 2020, Fig. 1B) determined the abdominal abscess was smaller than before and the abdominal wall soft tissue mass had remained the same size. There were multiple irregular nodules in the mesentery and multiple small lymph nodes behind the peritoneum. The patient ultimately underwent the operation, which found retroperitoneal masses, abdominal wall tumor, partial small bowel adhesion and intra-abdominal abscess. He underwent the resection of 4 retroperitoneal masses (the largest size 16 × 12 × 7.5 cm, and the remaining size 10 × 6 × 5 cm), abdominal wall tumor (10 × 8 × 4.5 cm) and partial small bowel with intestinal anastomosis and lysis of intestinal adhesion. The intra-abdominal abscess was also removed during the operation. The pathology of the tumor was spindle cell tumor with unclear borders, and it had invaded the surrounding fat and skeletal muscle tissue, involving the plasma layer and muscular layer of the partial small intestine (Fig. 2). Immunohistochemistry determined the tumor to be SMA (-), Caldesmon (-), β-Catenin nucleoplasm (+), S100 (-), SOX10 (-), CD34 (-), STAT6 (-), CD117 (scattered +), Dog-1 (-), and Ki-67 (+) for about 2%. After the operation, the patient was given parenteral nutrition and antibiotic therapy. He recovered well after the operation without abdominal pain or fever, and was discharged from the hospital in September 2020.

The patient had no evidence of recurrence on follow-up at 10 months. The Inflammatory indicators such as the white blood cell count, C-reactive protein level and serum procalcitonin were normal. An abdominal enhanced CT scan (July 2021, Fig. 1C) detected abdominal wall scars, high-density staples, and slight thickening of the anastomotic wall.

## 3 Discussion

Desmoid tumors (DTs), also known as aggressive fibromatosis, are a type of fibroproliferative disease caused by the monoclonal proliferation of fibroblasts. These tumors are rare. The incidence of DTs in FAP is 800- to 1000-fold higher than in the general population (0.03%).^[4]^ DTs occur in 10% to 15% of patients with FA, with a peak age of 30 to 40 years.^[5]^ Most patients are diagnosed as DTs after FAP. Our patient with a family history of FAP was diagnosed with FAP at the age of 31. He was diagnosed as DTs 2 years later, which was consistent with the literature.

The risks of developing DTs in patients with FAP include the presence of a family history, gene mutations, high estrogen levels, and surgical trauma.^[6]^ Most patients with FAP develop DTs within 5 years after surgery and 68% to 83% of patients develop DTs after abdominal surgery.^[7]^ It is speculated that iatrogenic trauma of the abdominal wall and abdominal pelvic cavity by surgery results in abnormal monoclonal proliferation of fibroblasts during tissue repair and reconstruction. Our patient underwent 2 abdominal surgeries and DTs developed 24 years after the appendectomy and 4 years after the cholecystectomy. The occurrence of DTs is considered to be related to abdominal surgery.

Similar to sporadic DTs, FAP-related DTs lesions are mainly in the abdominal cavity (about 80%), abdominal wall (10%–15%), and extra-abdominal (about 5%).^[2]^ Intra-abdominal DTs are mostly located in the mesentery. These tumors grow slowly, generally increasing by 2 to 9 cm per year, and locally without metastasizing distantly. Despite their benign nature, they can be infiltrative and multifocal, causing significant morbidity and mortality. They can invade the intestines and surrounding tissues, causing gastrointestinal bleeding, intestinal obstruction, and perforation. However, the intra-abdominal abscess formation in DTs is rare.^[3]^ There are only 8 case reports about degeneration of intra-abdominal DTs into abscess in literature (Table 1) thus far.^[3,8–14]^ Among 10 patients reported, 5 patients were diagnosed with FAP and 5 patients without FAP. The pathological mechanism about DTs complicated with abscess is not completely clear. Some researchers propose that mesenteric tumors compress the vasculature, leading to intestinal ischemia with bacterial transposition, causing subsequent necrosis and abscess formation. Fistulization to adjacent intestine can also result in abscess formation.^[8]^ In this case, the tumors were located in the abdominal wall and cavity. The tumor grew aggressively, invading surrounding fat, skeletal muscle tissue, and the serosal and muscular layers of the small intestine, resulting in local necrosis of the small intestine and mesenterium, and ultimately leading to an abdominal abscess.

**Table 1:** Summary of 8 case reports about desmoid tumors complicated by abscess.

Number	Report	Year	No. of patients	FAP related	1st treatment (no. of patients)	2nt treatment (no. of patients)	Follow-up time	Prognosis
1	Maldjian et al^[9]^	1995	3	3	Percutaneous drainage (3) + antibiotics (3)	Surgical resection (2)	None	
2	Cholongitas et al^[10]^	2006	1	None	Percutaneous drainage (1) + antibiotics (1)	Surgical resection (1)	2 yr	No recurrence
3	Ebrahimi-Daryani et al^[14]^	2008	1	None	Antibiotics (1)	Surgical resection (1)	None	
4	Peled et al^[11]^	2012	1	None	Antibiotics (1)	Surgical resection (1)	None	
5	Giovanni Alemanno et al^[8]^	2013	1	1	Percutaneous drainage (1) + antibiotics (1)		None	
6	Kai Huang et al^[12]^	2017	1	None	Percutaneous drainage (1) + antibiotics (1)		11 mo	No recurrence
7	M. Alam et al^[13]^	2020	1	1	Percutaneous drainage (1) + antibiotics (1)		6 wks	No recurrence
8	Omori et al^[3]^	2021	1	None	Antibiotics (1)	Surgical resection (1)	6 mo	No recurrence

FAP-associated with DTs was diagnosed based on clinical manifestation, surgical history, and imaging examination. CT and magnetic resonance imaging (MRI) scans can not only help for diagnosis, but also in determining the relationship between tumors and surrounding organs. The density of the lesions on CT imaging is uniform and an enhanced scan can show uniform enhancement. DTs had some specific features on MRI, including a stellar shape and extension into the fascial planes and fat tissue in a sunburst-like form. In addition, they are isointense on T1 and hyperintense on T2.^[15]^ Pathology is the gold standard for the diagnosis of DTs. Histological examination reveals paucicellular proliferation of fibroblasts and myofibroblasts in a dense collagenous background, spindle cells with small and regular nuclei, pale eosinophilic cytoplasm, and acellular central areas with increasing cellularity towards the periphery. Immunohistochemistry shows the cells are β-catenin, vimentin, Ki-67, SMA, CD68, and CD34 positive, which can assist with the diagnosis.^[16]^ The differential diagnosis of FAP associated with DTs includes metastatic tumors, lymphomas, tumors of mesenchymal origin, fibrosarcoma, and neurofibroma. In this case, an abdominal-enhanced CT scan showed a soft tissue mass in the abdominal wall and an irregular mesenteric soft tissue mass with internal fistula and abdominal abscess. FAP-related DTs was suspected and the biopsy confirmed the diagnosis.

Intra-abdominal DTs are commonly more difficult to resect compared with extra-abdominal and abdominal wall tumors. Surgery may be technically challenging, and the risk to the patient is high, particularly when these tumors occur in patients with FAP.^[17]^ For asymptomatic patients, close observation by serial imaging can be initiated with the interval of 3 to 6 months.^[18]^ For symptomatic patients, surgery and medical management are appropriate options, including non-steroidal anti-inflammatory drugs, estrogen antagonists, and chemotherapy drugs (e.g., doxorubicin and dacarbazine).^[19]^ Occasionally, surgical management becomes the only way in patients with complications, such as in this case where the patient developed abdominal abscess despite antibiotic treatment. In the majority of cases of DTs complicated with abscess reported, the first treatment consisted of antibiotic therapy with or without percutaneous drainage. The surgical resection of the tumor was the second approach due to persistent symptoms related to DTs. In addition, there were three patients successfully treated by antibiotic therapy and percutaneous drainage without surgery. The 4 patients reported with the follow-up time between 6 weeks and 2 years made a good recovery and had no recurrence. Our patient did not undergo percutaneous drainage for the abscess was not so large. However, he still had intermittent abdominal pain and fever after active anti-infective treatment. After 6 months, the blood routine examination was normal, and the abdominal abscess was smaller. He ultimately underwent the surgery. He was doing well on follow-up at 10 months.

## 4 Conclusions

In conclusion, we report the first case of FAP-related DTs initially presented with abdominal abscess in China. This case indicates that for patients with FAP who have a history of abdominal surgery and a progressively enlarging mass and abscess in the abdomen, it is necessary to consider the possibility of DTs. Seldom will FAP-related DTs degenerate into intra-abdominal abscess formation. CT and MRI of the abdomen and pelvis are feasible for further examination. Needle biopsy is helpful to confirm the diagnosis. Antibiotic therapy plus surgical resection of the tumor may be effective and make good prognosis.

## Author contributions

**Investigation:** Jun Wu.

**Writing – original draft:** Ailing Liu, Hua Liu, Xueli Ding.

**Writing – review & editing:** Zibin Tian, Tao Mao.
